# Blood Outgrowth Endothelial Cells Increase Tumor Growth Rates and Modify Tumor Physiology: Relevance for Therapeutic Targeting

**DOI:** 10.3390/cancers5010205

**Published:** 2013-02-18

**Authors:** Jonathan Pagan, Beata Przybyla, Azemat Jamshidi-Parsian, Kalpna Gupta, Robert J. Griffin

**Affiliations:** 1 Department of Radiation Oncology, University of Arkansas for Medical Sciences, 4301 West Markham Street, Little Rock, AR 72205, USA; E-Mails: bea.przybyla@gmail.com (B.P.); JamshidiAzema@uams.edu (A.J.-P.); RJGriffin@uams.edu (R.J.G.); 2 Vascular Biology Center and Division of Hematology-Oncology Transplantation, Department of Medicine, University of Minnesota Medical School, MN 72223, USA; E-Mail: gupta014@umn.edu

**Keywords:** endothelial precursor cells, HIFU, BOEC, tumor oxygenation, radiation response

## Abstract

Endothelial cell precursors from human peripheral blood have been shown to home to areas of neovascularization and may assist tumor growth by increasing or fortifying blood vessel growth. In the present study, the influence of these cells on tumor growth and physiology was investigated and the role of these cells as a therapeutic target or in determining treatment sensitivity was tested. After isolation from human blood and expansion *in vitro*, actively growing cells with verified endothelial phenotype (Blood Outgrowth Endothelial Cell, BOEC) were injected i.v. into tumor bearing mice for three consecutive days. The growth rate was significantly enhanced in relatively small RERF human lung tumors (*i.e*., less than 150 mm^3^) grown in immunocompromised mice by an average of 1.5-fold while it had no effect when injections were given to animals bearing larger tumors. There were no signs of toxicity or unwanted systemic effects. We also observed evidence of increased perfusion, vessel number, response to 15 Gy radiation and oxygenation in RERF tumors of animals injected with BOECs compared to control tumors. In addition, FSaII murine fibrosarcoma tumors were found to grow faster upon injection of BOECs. When FSaII tumors were subjected to a partial thermal ablation treatment using high intensity focused ultrasound (HIFU) there was consistently elevated detection of fluorescently labeled and i.v. injected endothelial precursors in the tumor when analyzed with optical imaging and/or histological preparations. Importantly, we also observed that BOECs treated with the novel anti-angiogenic peptide anginex *in-vitro*, show decreased proliferation and increased sensitivity to radiation. *In vivo*, the normal increase in FSaII tumor growth induced by injected BOECs was blunted by the addition of anginex treatment. It appears that endothelial precursors may significantly contribute to tumor vessel growth, tumor progression and/or repair of tumor damage and may improve the oxygenation and subsequent radiation response of tumors. We surmise that these cells are preferentially stimulated to divide in the tumor microenvironment, thereby inducing the significant increase in tumor growth observed and that the use of injected BOECs could be a viable approach to modulate the tumor microenvironment for therapeutic gain. Conversely, agents or approaches to block their recruitment and integration of BOECs into primary or metastatic lesions may be an effective way to restrain cancer progression before or after other treatments are applied.

## 1. Introduction

The growth and metastasis of tumors relies almost entirely on successful blood vessel formation and recruitment [1]. These processes, angiogenesis and vasculogenesis, respectively, are unique processes and mostly independent from one another. Angiogenesis is the process by which new blood vessels sprout from established vasculature, while vasculogenesis is specific for when endothelial and other endothelial precursors coalesce to form new postnatal blood vessels [2,3]. While evidence for the contribution of peripherally isolated BOECs in tumor vasculogenesis is present, its specific role is poorly understood [4,5]. Dudek *et al*. recently demonstrated the capability of using BOECs as a vehicle for successful, targeted delivery of anti-angiogenic compounds, while Kang *et al*. have recently shown BOEC-derived blood vessels capable of successful transplantation between mice [6,7]. This reinforces the importance of gaining a thorough understanding of BOECs’ role in tumor vasculogenesis, in order to continue developing new, targeted therapies and understand major mechanisms of tumor progression.

While isolation of BOECs and proving their existence has historically been a difficult task, it has been shown that it is possible to isolate bone marrow-derived BOECs from circulating blood [8,9]. These CD 31, CD34 and CD 45+ cells contribute to neoendotheliazation and neovascularization in postnatal organisms [10,11]. In the current study, we specifically refer to the cells as blood outgrowth endothelial cells (BOEC) due to their potential for mass expansion *in vitro* and specific distinction from circulating endothelial cells that expand modestly in culture. The ability of BOECs to expand under long term culture and maintain endothelial phenotype suggests great potential for utilization as therapeutic or detection vehicles in a variety of disease states [8]. Multiple studies have attempted to elucidate what factors are responsible for the migration of these cells to areas actively undergoing vasculogenesis, many of which have been focused on normal tissues [12,13]. Progress has been made, and it is accepted that VEGF plays a vital role in this migration and the incorporation of BOECs [14]. Studies by Zheng *et al*. [15], demonstrate SDF-1α concentration-dependent migration of BOECs, by using AMD3100 as an inhibitor, suggesting that SDF-1α, a chemokine that was originally isolated from bone marrow stromal cells and classified as a pre-B-cell growth stimulating factor, may play a role [16]. These studies, amongst others, provide a path for the development of therapeutics to block the recruitment of BOECs or purposeful delivery of these cells to create favorable conditions for treating or to assist the host in resolving disease states.

The goal of the studies presented here was to confirm the uptake and migration of exogenously administered BOECs to the tumor vasculature, and further understand their effects on the tumor microenvironment including whether or not an anti-angiogenic peptide could block their effects on tumor progression [2,3,4,5]. As high intensity focused ultrasound (HIFU) is being increasingly used in patient care, it presents itself as a useful and relevant tool in the study of damage repair in tumor tissues [17]. We investigated the role of BOECs immediately after sublethal ablation with HIFU, and their effect on radiation therapy response [18,19,20]. We present data showing that BOECs do migrate from the peripheral blood to sublethally ablated tumor. They can be targeted with an anti-angiogenic peptide and their effects on tumor physiology go beyond vasculogenesis, including increased tumor growth, increased oxygenation, as well as increased radiosensitivity.

## 2. Experimental Section

### 2.1. Tumor Cell Lines and Tumor Growth

Two cell lines were used in this study (FSaII and RERF). FSaII is a fibrosarcoma of C3H mice (Jackson Laboratories, Bar Harbor, MN, USA) which was originally obtained from Dr. Herman Suit (Massachusetts General Hospital, Boston, MA, USA), while RERF is a human non-small cell carcinoma purchased from ATCC (Manassas, VA, USA). While FSaII was derived from C3H mice, all BOEC injections took place in nude (nu/nu) mice to avoid an immune response. Stock cells are stored in liquid nitrogen and new cultures are established every 2–3 months. Both tumor cells grow well in RPMI-1640 medium supplemented with 10% bovine calf serum.

Tumor induction: Tumor cells in exponential growth phase were harvested using 0.25% trypsin in Hepes buffered medium, washed and counted. A subcutaneous injection of 2 × 10^5^ cells in 0.05 mL serum-free medium was made in the hind thigh of female C3H mice (FSaII) or 4 × 10^6^ in nu/nu mice (RERF).

### 2.2. Culture of Endothelial Cells and Injection

**Figure 1:**
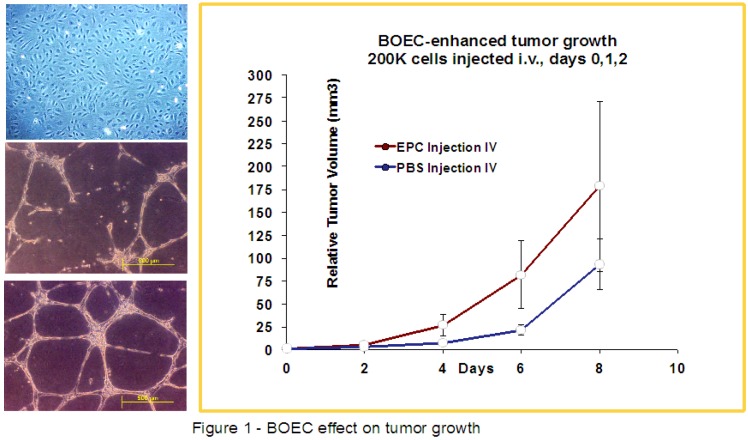
BOEC have cobblestone growth pattern, form tubules and are responsive to VEGF-A. Left panels, top; typical morphology of BOECs in near confluent culture at passage 5, middle; tubule formation in BOEC on growth factor reduced Matrigel in response to PBS, bottom; BOEC s are responsive to VEGF165 at 100 ng/mL for 24 h forming more extensive and mature tubules. RIGHT panel: C3H mice were inoculated with FSaII tumors, and then injected with 2 × 10^5^ BOECs on days 0, 1 and 2. When compared to controls, the tumors exposed to exogenous BOEC by three daily i.v. injections during early growth demonstrated higher final tumor volume (*p* < 0.09 on days 6–8). A difference in relative size of 93.26 mm^3^ to 178.5 mm^3^ was seen eight days post injection with BOECs.

The cells used for these studies were supplied by the laboratories of Drs. Robert Hebbel at the University of Minnesota (Minneapolis, MN, USA) and Joyce Bischoff at Harvard University/Beth Israel Medical Center (Boston, MA, USA) using similar techniques for isolation and *ex vivo* expansion [21,22]. The cells obtained from the Hebbel and Bischoff were extensively verified to have an endothelial phenotype (CD31+, CD45−, CD14−, vWF+ and VE-Cadherin+) in the respective laboratories and were used by our team within two passages from the stock cells [8,22]. A picture of typical morphology of the BOECs at initial plating as well as evidence for tube formation after culture with PBS or VEGF165 at 100 ng/mL for 24 h on growth factor reduced Matrigel, and human BOEC in culture at passage 5, is shown in Figure 1. Through methods described previously, aliquots of cells were obtained from each collaborating lab, thawed and cultured in tissue culture flasks coated with 1% gelatin in EGM-2 full endothelial cell medium [23]. Briefly, cells were expanded and passed 2–3 times until enough cells were present for harvest and inoculation. After trypsinizing and washing, actively growing cells with verified endothelial phenotype were collected and injected in desired numbers in buffered saline i.v. into tumor bearing mice for up to three consecutive days.

### 2.3. HIFU-Ablation of FSaII Tumors Followed by BOEC Injection

Immunocompromised mice (used to improve optical imaging results by avoiding fur artifacts) were injected with FSaII tumor cells in the right rear leg. Tumor ablations were performed on 8 mm diameter tumors using a spherically-focused ultrasound applicator specially designed for heating small animals. The transducer was run at 40 W with a variable duty cycle to control heating. The tumors were ablated at an average peak temperature of 60 °C for 1.5 min. One group of mice was immediately injected IV with near infrared emitting indocyanine green (ICG)-labeled BOECs, while the other group was injected with BOECs labeled with a stable *in situ* dye (PKH). The mice injected with ICG-labeled BOEC were optically imaged at various time points up to 72 h. These images were then compared to those of untreated tumors to observe the migration patterns of BOEC in mice that had received HIFU ablation. Tumor and tissue samples were taken from the mice injected with PKH labeled BOEC cells 24 h post ablation and frozen for histological examination of BOEC incorporation into tumor and various normal tissues.

The dual-laser targeting of the HIFU transducer allowed for accurate placement of the focus, and therefore accurate and reproducible tumor ablations. We routinely ablated approximately 50% of tumor tissue, leaving the other 50% peripheral tumor tissue viable.

### 2.4. Oxygenation Measurement

Tumor oxygenation was measured using and oxylab fiberoptic system (Oxford Optronix, Ltd., Oxford, UK). The probe was inserted via a needle track to approximately 7 mm deep in the tumor. After the reading stabilized (approximately 2–3 min in most cases, the mmHg value was recorded and the probe was then retracted 2 mm, allowed to stabilize, and another reading was taken. A third reading was taken after an additional 2 mm retraction of the needle. Values were averaged from 3–5 tumors per group.

### 2.5. Radiation Therapy

Radiation was applied locally using a 250 kV orthovoltage system (Philips Medical Systems, Brookfield, WI, USA) as described previously [24,25]. Briefly, animals were anesthetized, covered with a lead sheet and the tumor was gently placed outside of the shielding by taping or tying the foot and then exposed to X-rays at a dose rate of 1.4 Gy/min.

### 2.6. Statistical Analysis

Student’s t-test was used to compare the difference of the means in the cell survival and tumor volume related studies, with an arbitrary significance set at *p* < 0.5. For the tumor oxygenation measurements, a Mann-Whitney test was employed.

## 3. Results

### 3.1. Injected BOECs Alter Tumor Oxygenation and Increase Tumors Response to Radiation

Our initial studies focused on tumor growth rate changes after BOEC i.v. injections during the early period of tumor development in mouse models. In Figure 1, the morphology and responsiveness of the BOECs that we typically worked with are shown, as well as a tumor growth plot of FSaII fibrosarcomas growing in mice inoculated with 2 × 10^5^ BOECs on the first three days after tumor cell inoculation. There was a noticeable acceleration of tumor growth within the first 4 days that was most apparent at day 6 after tumor inoculation. 

**Figure 2:**
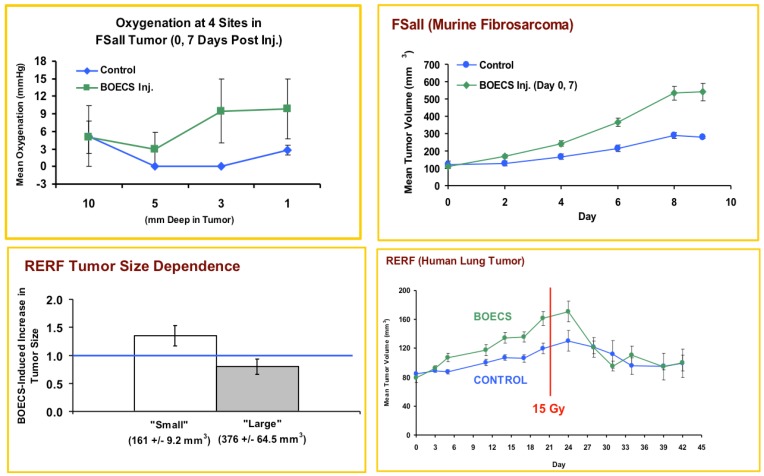
TOP PANELS: BOEC were injected i.v. at Day 0 and Day 7 (2.5 × 10^5^ cells/injection). At Day 8, tumor oxygenation was assessed in control (n = 3) and BOEC (n = 3) treated mice using a fiberoptic probe (Oxylab, Oxford Optronix, Oxford, UK); ns difference due to low sample #. The tumor growth was found to significantly increase in the tumors of mice injected with BOECs (*p* < 0.01 on days 4–9. BOTTOM: RERF human tumor xenografts were injected on Day 0, 1 and 2 with 2.5 × 10^5^ cells. The left panel illustrates the effect of injected BOECs on smaller *vs*. larger tumors at the time of injection (*p* = 0.05 for large *vs*. smaller tumors), and the right panel show the growth curve of the tumors, including after a single exposure of the tumor to 15 Gy on Day 21 (*p* < 0.05 between control and BOEC-injected animals on days 18 and 24). The tumors treated with BOECs regressed more than the control tumors after radiation.

We subsequently observed a trend for increased tumor oxygenation profiles in FSaII tumor-bearing mice that received BOEC injections on Day 0 and Day 7 after tumor inoculation, as shown in Figure 2. In addition, FSaII tumors in mice injected with BOECs were found to be larger than tumors in control mice by day 9 of tumor growth. Due to our interest in the therapeutic consequences of increased BOEC presence in tumor bearing mice, we then performed a radiation response study in RERF human lung cancer xenografts. A single radiation dose of 15 Gy administered when BOEC-treated animals had a substantially larger tumors than control mice (day 42) induced a 1.65-fold decrease in RERF tumor volume in BOEC-treated mice as compared to only a 1.18-fold decrease in control RERF tumors (Figure 2, bottom panels). This increased therapeutic response to ionizing may have been partially due to an increase in tumor oxygenation (as shown in Figure 2) even though the tumor size in the BOEC exposed mice was larger than control mice at the time of irradiation. 

### 3.2. Anginex Antagonizes Vasculogenesis Stimulated by BOECs and Increases BOEC Susceptibility to Radiation

**Figure 3:**
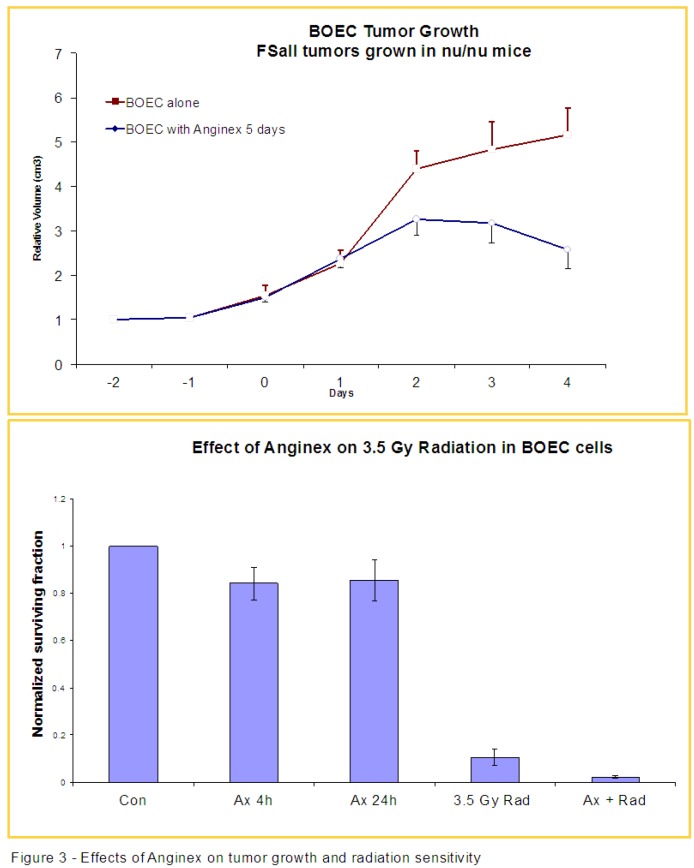
TOP: Nu/nu mice were inoculated with FSaII tumor and received daily i.v. injections of either BOECs or BOECs with anginex. The relative volumes were plotted, and the tumors in the mice that received both BOEC and anginex did not demonstrate the increased tumor volume seen in the mice that received only BOECs injection (*p* < 0.013 on day 4 for BOEC injected *vs*. BOEC injected, anginex treated mice). BOTTOM: BOECs grown *in-vitro* were exposed to anginex and monitored (colonies counted) for twenty-four hours. The plates exposed to anginex and the controls were then given 3.5 Gy of radiation and their cell colonies counted. The BOECs exposed to anginex demonstrated increased sensitivity to radiation, represented by a greater decrease in the number of colonies after radiation exposure than in radiation alone injection (*p* = 0.06 for anginex treated + radiation *vs*. radiation alone).

We previously demonstrated that anginex, a designed peptide originally named βpep-25, increases tumor sensitivity to radiation in animal models due to its anti-angiogenic properties [25]. The creation of anginex involved using basic folding principles and incorporating short sequences from the beta-sheet domains of anti-angiogenic proteins, and is detailed in the original paper from Griffioen *et al.* [26]. Here we observed that anginex treatment i.v. blunts the angiogenic/tumor growth-promoting effects of exogenous BOECs injected into mice early (days 0, 1 and 2) in tumor growth, resulting in smaller tumors (Figure 3). After introducing exogenous BOECs, we have found that tumors consistently grow to larger sizes. When the growth rates of tumors exposed to exogenous BOECs compared to those treated with both BOECs and anginex, we observed that the antiangiogenic effects of anginex exert their effects through a pathway that includes blocking at least part of the activity/viability of BOECs. *In-vitro* studies have shown that anginex alone decreases the number of surviving BOEC colonies after a 4 h exposure by about 20%. Importantly, we also noted an increased sensitivity to radiation in BOECs pre-treated with anginex, with a decrease of BOEC viability of nearly 90% in the combination treatment group (Figure 3).

### 3.3. Injected BOECs Migrate to Tumors after Thermal Ablation

**Figure 4:**
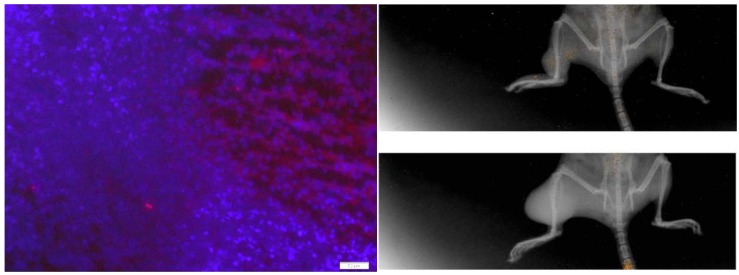
Nu/nu mice were inoculated with FSaII tumor unilaterally in the rear extremity and allowed to grow for 7–10 days. After receiving HIFU ablation, 2 × 10^5^ BOECs labeled with either PKH (LEFT) or ICG (RIGHT), were injected i.v. through the dorsal tail vein. LEFT: FSaII tumors examined histologically 24 h after injection with BOEC labeled with PKH demonstrates the presence of BOEC in tumors after HIFU ablation. Blue-Tumor, Red- labeled BOECs surrounded by a box. RIGHT TOP: 72 h post HIFU ablation and injection with ICG-labeled BOEC optically imaged using a Kodak imaging apparatus. RIGHT BOTTOM: The control group 72 h post injection of ICG-labeled BOEC optically imaged using the Kodak machine. The control group demonstrates significantly fewer BOEC cells than tumors treated with HIFU.

After local thermal ablation, tumor recurrence is common and likely reliant on angiogenesis. Following the above studies showing that BOECs migrate to untreated tumor and contribute to significant vasculogenesis and tumor growth, we investigated thermal ablation as a way to maximize tumor release of chemokines to attract BOECs. Immediately after injection, a majority of the ICG labeled BOEC cells were detectable in the lung tissue bilaterally (data not shown). Over the course of 72 h their concentration in lung tissue decreased steadily, so that at 72 h there was no discernable presence remaining in the lungs. However, BOECs fluorescently-labeled with PKH were detectable in the surviving tumor 24 and 72 h post ablation with HIFU, respectively (Figure 4). This data presents new evidence that sublethally ablated tumors signal for BOECs to migrate from the periphery to the surviving tumor bed. The areas of noted labeled cell accumulation are boxed in the images.

## 4. Discussion

Overall, the results we have observed in two tumor types using two distinct, but similar sources of human BOECs, demonstrates at least three paths with potential for development of cancer therapies exploiting or targeting peripheral blood endothelial precursors cells. First, we demonstrate that injected BOECs early in tumor development consistently increase the final size of the tumor (Figure 2, Figure 3). This suggests that elucidating the exact cellular mechanisms responsible for BOEC migration, be it VEGF, SDF-1α or other factors, will potentially allow blocking this migration leading to smaller, less virulent tumors [13,15]. For example, anginex, a β-sheet-forming peptide, has potent *in vivo* antiangiogenic activity [27,28]. In a previous study in our lab, we found that anginex acts a potent endothelial radiosensitizer and we demonstrated that combining this compound with radiation therapy may lead to improved clinical outcomes [29]. These observed clinical outcomes could possibly be attributed to the increased sensitivity to radiation demonstrated by BOECs in the presence of anginex demonstrated in Figure 1. A study by Dings *et al*. in conjunction with our results, demonstrates the promise for endothelial targeted therapy as an adjuvant to radiation therapy. Further, our *in-vitro* data suggests that the direct radiation and drug sensitivity of BOECs may be reduced relative to that of normal cells or tumor cells. Therefore, the increased tumor burden induced by elevated numbers of BOECs in the blood stream in our current study, may in fact argue for the earlier use of chemotherapeutics which block the migration of BOECs to the tumor site. 

Once the BOECs are at the tumor site, they may contribute to resistance due to their inherent treatment resistance—similar to studies demonstrating that hematological or other stem-like cells are typically resistant to drug or radiation therapy relative to their more differentiated counterparts [30,31]. A recent study by Taylor *et al*. found that equally important to early use of vascular-disrupting agents (VDA), is considering a potential second burst, or rebound, of BOECs that occur later in the disease course potentially conferring VDA resistance [32]. Conversely, since our data also shows that the presence of the migrated BOECs may be able to increase tumor oxygenation and subsequent radiation response, we will be concurrently exploring whether the deliberate introduction of these vasculogenic compounds may in fact enhance current radiotherapeutic techniques and/or allow useful delivery of gene therapy or other approaches [6]. Importantly, a very clear understanding of these contradictory effects of BOECs is necessary before a rational plan to exploit them in combination with current treatment methods can be implemented. Given that anginex increases BOECs sensitivity to radiation, this may represent one possible way of counteracting the natural recruitment of BOECs in response to tumor progression or therapy.

Second, our data suggest BOECs may provide a route, or be an important variable, to improving response to radiation or other therapies. Chemical radiosensitizers have been investigated for many years, ranging from traditionally used chemotherapeutic agents such as 5-FU, to radiosensitizer-coated gold fiducials [33,34]. Our discovery of increased radiation sensitivity in tumors treated with BOEC injections early in tumor growth putatively due to an improvement of tumor oxygenation is a proof of concept for involving biological stem cell radiosensitizing strategies. It has been known for some time that hypoxic cells induce radiation resistance [35]. Over the last 50 years many compounds have been investigated for their potential to increase tumor oxygenation. More recently, the importance of nitric oxide has been explored, and exciting findings involving the production of inducible nitric oxide synthase by macrophages have been reported [36]. BOECs represent another possible alternative to more traditional radiosensitizers. The apparent contradiction between increased tumor growth rate induced by BOECs and improved radiation sensitivity via improved physiology highlights the need for rational design of any treatment or delivery approach to be developed around the targeting of tumors with naturally occurring BOECs or those introduced by infusion. In addition, the use of subcutaneously injected tumors, and some with rapid growth, in the current study may have introduced artificial results owing to the known fact that inoculated tumors do not grow like spontaneous neoplasms in various host tissues. Spontaneous malignancies in animals and humans utilize the stromal cells around them and recruit cells such as BOECs in distinct and unique ways. A step towards better understanding of these deficiencies may be to use orthotopic models, and eventually transgenic models where spontaneous tumor growth occurs.

Lastly, we have observed that in tumors that undergo traumatic tissue damage (such as might occur after surgery or in our case, thermal ablation procedures) the remaining microenvironment is conducive to the migration of bone marrow derived BOECs. This response has important therapeutic implications and likely influences the ability of tumors to recur and or metastasize—a known issue with thermal ablative approaches to date. While performing therapeutic HIFU ablation, the goal is always to destroy the entire tumor burden, however, even in the event that complete, or nearly complete, tumor ablation is obtained, granulation tissue, along with other inflammatory cells remain at the periphery [37]. Other types of thermal ablation or surgical excision methods may induce differing patterns and extent of BOEC mobilization and recruitment depending on the degree and temporal characteristics of the inflammatory response. While chemotherapy and radiation are already being used as adjuvant therapy to HIFU, there is a need for novel targeted therapeutics as well. The recruitment of BOECs may be an important variable in the development of therapies to enhance the already promising future of HIFU [38].

## 5. Conclusions

Naturally occurring or exogenously introduced endothelial-precursor cells from the peripheral blood appear to be a realistic medium for cell therapy-based anti-cancer approaches. We have demonstrated a role for BOECs in increasing tumor growth rate, the ability to radiosensitize BOECs with anti-angiogenic agents, a BOEC-induced increase in tumor oxygenation and BOEC recruitment after non-invasive tumor thermal ablation. These results suggest multiple targeting approaches and relevant information to control or exploit tumor vasculogenesis to the benefit of patients.
